# Neutrophil Extracellular Traps Promote Urolithiasis Formation in Dogs: A Preliminary Study

**DOI:** 10.3390/ani16060942

**Published:** 2026-03-17

**Authors:** Hao Shi, Ruizi Ren, Liwei Zeng, Yiwen Zhang, Wenkai Zhang, Meilin Qiao, Yipeng Jin

**Affiliations:** 1 College of Veterinary Medicine, China Agricultural University, Beijing 100193, China; 2 China Agricultural University Veterinary Teaching Hospital, Beijing 100193, China

**Keywords:** neutrophil extracellular traps, dog, urolithiasis, struvite, calcium oxalate

## Abstract

Canine urolithiasis is a common and highly recurrent urinary tract disease, with struvite and calcium oxalate being the predominant stone types. Stone formation has traditionally been attributed to urinary physicochemical factors, including urine pH, mineral supersaturation, and urinary tract infection. However, these factors alone cannot fully explain the persistent growth and rapid progression of stones in affected dogs. In this study, we provide evidence that innate immune mechanisms, particularly neutrophil extracellular traps (NETs), are associated with canine urolith formation. We found that neutrophils, key cells of the innate immune system, release neutrophil extracellular traps composed of DNA and antimicrobial proteins, which are consistently present within urinary stones and their surrounding microenvironments. Common canine urinary pathogens were shown to trigger this response, and these immune-derived structures promoted crystal nucleation, aggregation, and stone growth in experimental systems. Importantly, enzymatic degradation of NETs by DNase I attenuated NET-associated stone growth under in vitro conditions. These findings suggest that canine urinary stones develop not only as a consequence of physicochemical factors, but also in association with inflammation-driven biomineralization processes involving NETs. Recognizing the contribution of innate immunity provides new insight into stone recurrence and may inform future preventive and therapeutic strategies.

## Simple Summary

Urinary stones are common in dogs and often return after treatment. The most common types are struvite and calcium oxalate stones. Stone formation has traditionally been explained by changes in urine acidity, high mineral concentrations, and urinary tract infections. However, these factors cannot fully explain why stones continue to grow and recur in many dogs. In this study, we explored whether the dog’s immune system may also play a role in this process. Certain immune cells can release web-like structures made of deoxyribonucleic acid and antimicrobial proteins to trap invading microorganisms. We found that these structures were present within urinary stones and in the surrounding environment. In laboratory experiments, common bacteria from canine urinary tract infections triggered the formation of these structures, which then promoted the formation and growth of mineral crystals. When these structures were broken down using a specific enzyme, stone growth was reduced. These findings suggest that urinary stone formation in dogs may be linked not only to chemical changes in urine but also to inflammation-related processes. Understanding this mechanism could help to explain why stones recur and may support the development of new prevention and treatment strategies.

## 1. Introduction

Urolith formation is initiated by urinary supersaturation of mineral ions, followed by crystal precipitation and progressive local deposition, ultimately leading to the formation of macroscopic uroliths [1]. Urolithiasis is one of the most common urinary tract disorders in dogs. Among the various urolith types, struvite and calcium oxalate (CaOx) represent the two predominant mineral compositions in canine patients [2,3]. The pathogenesis of these two urolith types differs in several key aspects [4].

Canine struvite urolithiasis is typically associated with urinary tract infection (UTI) [4]. Urease-producing bacteria, most commonly *Staphylococcus* spp. and *Proteus* spp., are the principal microbial drivers of struvite formation [5,6,7]. These organisms hydrolyse urea to generate ammonium and bicarbonate ions. Ammonium subsequently combines with urinary magnesium and phosphate to form magnesium ammonium phosphate (MAP) crystals, whereas the accumulation of bicarbonate alkalinises urine, thereby further decreasing MAP solubility and promoting crystal precipitation and deposition [6,8,9]. In addition, bacterial infection facilitates stone formation by increasing bacterial adhesion to the positively charged crystal faces of MAP [10]. Consequently, when infection persists, struvite uroliths can develop within a relatively short period and continue to enlarge during the course of infection [9]. Clinically, dogs with infection-associated urolithiasis also exhibit higher rates of stone recurrence [11].

In contrast, the formation of CaOx uroliths is primarily attributed to urinary supersaturation with calcium and oxalate ions [12]. Established risk factors include hypercalciuria, hyperoxaluria, an acidic urinary environment and decreased concentrations of crystallisation inhibitors [13,14,15]. In addition, metabolic disorders, genetic predisposition, metabolic acidosis, dietary composition and alterations in the intestinal oxalate-metabolising microbiota may disrupt calcium–oxalate homeostasis and thereby increase the risk of CaOx urolith formation [12,16,17]. However, recent studies analysing the relationship between urolith composition and urinary tract pathogens have suggested that non-urease-producing bacterial infections may also be associated with CaOx urolithiasis, although the underlying mechanisms remain unclear [18].

Urolith formation is a complex biological process [19]. It is not merely the result of inorganic mineral precipitation driven by urinary chemical imbalance, but also involves regulatory mechanisms mediated by multiple organic components [20]. However, most of these mechanisms remain incompletely characterised [21]. In addition, current evidence does not adequately explain why infection-associated uroliths can develop within shorter time frames [22]. These observations suggest that additional, as yet insufficiently recognised biological regulatory mechanisms participate in urolith formation and pathological mineralisation.

Under physiological conditions, neutrophils function as “tissue-patrolling cells” and are widely distributed throughout body tissues [23]. When inflammation or infection develops within the urinary tract, neutrophils act as frontline effector cells of innate immunity [24]. Under the regulation of multiple pro-inflammatory mediators, they are rapidly recruited and accumulated to participate in host defence responses [25,26]. Previous studies have demonstrated that neutrophils can be activated by a wide range of pathogen-associated molecular patterns (PAMPs) and damage-associated molecular patterns (DAMPs), leading to the formation of neutrophil extracellular traps (NETs) [27].

NETs were first described by Brinkmann et al. in 2004 as web-like structures composed of chromatin DNA decorated with histones, granular and cytoplasmic proteins [28]. NETs were initially recognised to have the ability to trap and eliminate invading pathogens [28]. NETs are now regarded as a double-edged sword [29]. On one hand, pro-inflammatory mediators released during NET formation can induce local inflammatory responses [30]. These mediators can also directly damage host tissues [31]. On the other hand, excessive NET accumulation leads to the formation of aggregated NETs (aggNETs). These structures contribute to the development of various occlusive diseases [32]. In recent years, aggNETs have also been shown to participate in the mineralisation of several stone-related disorders, including gouty tophi, gallstones and dental calculus [33,34,35].

In the context of urolithiasis, upregulation of NET-associated proteins has been reported in infectious matrix stones [22]. However, direct evidence supporting a structural role of NETs in urinary stone formation remains limited. These observations raise a key question: in canine urolithiasis, do NETs constitute a structural component of stone mineralisation and thereby promote rapid stone formation and progression?

However, whether NETs represent an active structural component of stone formation in dogs has not been experimentally addressed. To answer this question, the present study employed ELISA and immunofluorescence co-localisation analyses to confirm the presence of NETs within canine struvite and calcium oxalate uroliths and their corresponding formation microenvironments. Co-culture experiments with two common uropathogenic bacteria demonstrated that urinary tract pathogens may represent important drivers of NET formation and may increase the risk of stone recurrence. Furthermore, by establishing in vitro mineral ion systems and performing crystal nucleation and stone growth assays, we further demonstrated that the NET-derived DNA scaffold functions as an organic matrix that promotes crystal nucleation and aggregation, facilitates crystal adhesion to the stone surface, and thereby accelerates stone growth.

Collectively, these observations support the concept that inflammatory mechanisms may contribute to urolith formation in parallel with established physicochemical processes.

## 2. Materials and Methods

### 2.1. Animals and Sample Sources

Healthy dogs (*n* = 16), dogs with struvite urolithiasis (*n* = 8) and dogs with calcium oxalate urolithiasis (*n* = 8) were recruited from the Teaching Animal Hospital of China Agricultural University. All enrolled dogs underwent comprehensive clinical evaluation, including complete blood count (CBC), serum biochemical analysis, routine urinalysis and diagnostic imaging (ultrasonography or radiography). Dogs presenting with severe systemic disease, hemorrhagic disorders, acute infectious disease or other conditions that could interfere with experimental outcomes were excluded. Healthy dogs were confirmed to be free of urinary tract disease and had not received antibiotics within one month prior to enrolment. Dogs with urolithiasis underwent cystotomy, and urolith composition analysis and bacterial culture were performed. Culture samples were obtained from bladder mucosa or preoperative cystocentesis urine. Infectious urolithiasis was defined as bacterial growth in these samples. Non-infectious urolithiasis was defined as the absence of bacterial growth in dogs that had not received antimicrobial therapy within one month prior to sampling. Bacterial identification was conducted by the hospital clinical microbiology laboratory according to standard diagnostic procedures.

Urine samples were collected from healthy dogs (*n* = 16) and dogs with urolithiasis (struvite, *n* = 8; calcium oxalate, *n* = 8). Urolith specimens were obtained from the same 16 dogs with urolithiasis. Blood samples used for in vitro experiments were collected from healthy donor dogs (*n* = 5) owned by hospital staff.

*Staphylococcus pseudintermedius* and *Proteus mirabilis* were provided by the China Agricultural University Veterinary Teaching Hospital.

All procedures were approved by the Institutional Animal Care and Use Committee (IACUC) of China Agricultural University, and all animal-related experiments were conducted in accordance with local regulations and institutional ethical guidelines.

### 2.2. Sample Collection and Processing

Freshly retrieved uroliths were rinsed with sterile physiological saline and temporarily stored at 4 °C. A subset of each specimen was directly fixed for paraffin embedding, whereas the remaining portions were snap-frozen in liquid nitrogen and stored at −80 °C for subsequent in vitro mineralisation assays.

Urine samples were centrifuged within 2 h of collection (4 °C, 2000 rpm, 20 min). The supernatants were aliquoted, snap-frozen in liquid nitrogen and stored at −80 °C for subsequent quantification of MPO–DNA complexes.

Whole blood was collected from the jugular vein of healthy donor dogs into sodium citrate-containing anticoagulant tubes (8–10 mL per collection) and processed within 2 h to ensure optimal cell separation efficiency.

### 2.3. Quantification of Urinary MPO–DNA Complexes by ELISA

Urinary concentrations of MPO–DNA complexes were quantified using a canine-specific MPO–DNA ELISA kit (MM-85344O1; Enzyme Immunoassay Co., Nanjing, China) according to the manufacturer’s instructions. Each urine sample was analysed in triplicate, and the mean value was used for statistical analysis. Optical density (OD) was measured at 450 nm using a microplate reader (Spark, TECAN, Grödig, Austria). MPO–DNA concentrations (ng/L) were calculated based on the standard curve generated for each assay.

### 2.4. Preparation of Urolith Paraffin Sections and Immunofluorescence Staining

Fresh canine struvite (*n* = 6) and calcium oxalate (*n* = 6) uroliths were rinsed with sterile distilled water and fixed in 4% paraformaldehyde for 48 h. Fixed specimens were decalcified in Ethylene Diamine Tetraacetic Acid (EDTA) decalcification solution (pH 7.2) for 3–4 days until adequately softened. Following decalcification, uroliths were routinely paraffin-embedded, sectioned and deparaffinised, after which antigen retrieval was performed.

Sections were blocked with 5% bovine serum albumin (BSA) at 37 °C for 1 h and then incubated overnight at 4 °C with primary antibodies diluted in 1% BSA: rabbit anti-citrullinated histone H3 (Cit-H3; ab5130, Abcam, Cambridge, UK; 1:200) or rabbit anti-neutrophil elastase (NE; bs-23549R, Bioss, Beijing, China; 1:200). Negative control sections were incubated with PBS. After washing with PBS, sections were incubated with FITC-conjugated goat anti-rabbit secondary antibody (AS011, Abclonal, Wuhan, China; 1:100) for 1 h at room temperature in the dark. Extracellular DNA was stained with propidium iodide (10 μg/mL; C0080, Solarbio, Beijing, China; 1:100) for 10 min. After PBS washing, sections were mounted using an anti-fade mounting medium and fluorescence images were acquired using a laser scanning confocal microscope (A1HD25, Nikon, Tokyo, Japan).

### 2.5. Isolation and Preparation of Canine Peripheral Blood Neutrophils

Canine peripheral blood neutrophils were isolated using a commercial neutrophil isolation kit (LZS1087; TBDscience, Tianjin, China) according to the manufacturer’s instructions. Briefly, 5 mL of separation solution 1 and 2 mL of separation solution 2 were sequentially added to a sterile 15 mL centrifuge tube, followed by careful layering of 2–3 mL of freshly collected canine peripheral blood. Samples were centrifuged in a horizontal rotor centrifuge (Thermo Scientific, Waltham, MA, USA) at 600× *g* for 30 min at 20 °C. Cells in the second white interface layer were collected and resuspended in 10 mL PBS, followed by centrifugation at 250× *g* for 10 min at 20 °C. After discarding the supernatant, residual erythrocytes were lysed using red blood cell lysis buffer and the suspension was centrifuged at 300× *g* for 5 min at 4 °C. The supernatant was discarded, and cells were washed once with PBS. Finally, neutrophils were resuspended in 1–2 mL Roswell Park Memorial Institute 1640 (RPMI-1640) medium to obtain a neutrophil suspension for subsequent experiments.

### 2.6. Preparation of Bacterial Suspensions

Frozen stocks of *S. pseudintermedius* and *P. mirabilis* were streaked onto brain–heart infusion (BHI) agar plates and incubated at 37 °C for 24 h. Single colonies were then inoculated into 10 mL BHI broth and cultured at 37 °C with shaking at 200 rpm overnight to reach the logarithmic growth phase. Bacterial numbers were estimated using the plate counting method.

Bacterial cultures were washed twice with PBS. *S. pseudintermedius* was centrifuged at 4000× *g* for 5 min at 4 °C, whereas *P. mirabilis* was centrifuged at 6000× *g* for 10 min at 4 °C. After discarding the supernatant, bacterial pellets were resuspended in RPMI-1640 medium to prepare bacterial suspensions for subsequent experiments.

### 2.7. In Vitro Induction and Degradation of NETs

Isolated neutrophils were seeded into 24-well culture plates at a density of 5 × 10^5^ cells per well and cultured in RPMI-1640 medium supplemented with 10% fetal bovine serum (FBS). Cells were incubated for 30 min at 37 °C in a humidified atmosphere containing 5% CO_2_ prior to stimulation. Neutrophils were then assigned to the following treatment groups: (1) PMA group, in which NET formation was induced with 400 nM phorbol 12-myristate 13-acetate (PMA; MCE, Monmouth Junction, NJ, USA); (2) control group, in which an equal volume of PBS was added; and (3) PMA + DNase I group, in which cells were treated with 400 nM PMA in combination with 100 U/mL DNase I (EN401-01, Vazyme, Nanjing, China) to enzymatically degrade NETs. Following 4 h of incubation, cells were used for subsequent biomineralisation assays. The concentration of PMA was selected based on preliminary dose–response experiments.

### 2.8. Bacterial Stimulation of Neutrophils

Isolated neutrophils were seeded into 24-well culture plates at a density of 5 × 10^5^ cells per well or into black-walled, clear-bottom 96-well plates at a density of 1 × 10^5^ cells per well and cultured in RPMI-1640 medium. After incubation for 30 min at 37 °C in a humidified atmosphere containing 5% CO_2_, cells were allocated to the following treatment groups: (1) PMA group, in which neutrophils were stimulated with 400 nM PMA; (2) bacterial stimulation groups, in which *S. pseudintermedius* or *P. mirabilis* was added at a multiplicity of infection (MOI) of 1000; and (3) control group, in which an equal volume of RPMI-1640 medium was added. After 4 h of incubation, cells were subjected to immunofluorescence staining or Sytox Green staining for NET detection.

### 2.9. Immunofluorescence Staining of Neutrophils

Following the treatments described in Section 2.7 and Section 2.8, culture supernatants were removed and cells were fixed with 4% paraformaldehyde at room temperature for 20 min. After washing with PBS, cells were blocked with 5% bovine serum albumin (BSA) at 37 °C for 1 h and then incubated overnight at 4 °C with rabbit anti-citrullinated histone H3 (Cit-H3; ab1503, Abcam, Cambridge, UK; 1:200) diluted in 1% BSA. Negative control wells received PBS only. After PBS washing, cells were incubated with FITC-conjugated goat anti-rabbit secondary antibody (AS011, Abclonal, Wuhan, China; 1:100) for 1 h at room temperature in the dark. Extracellular DNA was stained with propidium iodide (10 μg/mL; C0080, Solarbio, China; 1:100) for 10 min. Cells were washed with PBS, mounted using an anti-fade mounting medium, and images were acquired using an immunofluorescence microscope (Olympus, Tokyo, Japan).

### 2.10. Sytox Green Assay for Extracellular DNA Release

Following the treatments described in Section 2.8, extracellular DNA release was quantified using a Sytox Green assay kit (also named Nuclear Green assay kit; C1181, Beyotime, Shanghai, China). Briefly, 25 μL of 1× Sytox Green working solution diluted in Assay Buffer was added to each well and incubated at 37 °C for 20 min. Fluorescence was measured using a microplate reader (Spark, TECAN, Grödig, Austria) at excitation/emission wavelengths of 485/525 nm to assess the level of extracellular DNA release.

### 2.11. Preparation of Mineral Ion Solutions

Calcium chloride dihydrate (CaCl_2_·2H_2_O), sodium oxalate (Na_2_C_2_O_4_), magnesium chloride hexahydrate (MgCl_2_·6H_2_O), ammonium chloride (NH_4_Cl), sodium dihydrogen phosphate (NaH_2_PO_4_) and ammonia solution were purchased from Macklin (Shanghai, China). All reagents were prepared as 100 mM stock solutions in 0.9% sodium chloride. All subsequent dilutions and reaction solutions were prepared using 0.9% sodium chloride as the solvent.

To establish the CaOx crystallisation system, the 100 mM CaCl_2_·2H_2_O stock solution was diluted to 10 mM, and the 100 mM Na_2_C_2_O_4_ stock solution was diluted to 1 mM. Both solutions were adjusted to pH 7.0, filtered through 0.22 μm membranes and sterilised by ultraviolet irradiation prior to use.

To establish the MAP crystallisation system, 100 mM stock solutions of MgCl_2_·6H_2_O, NH_4_Cl and NaH_2_PO_4_ were mixed at a volume ratio of 1:1:1:22. The resulting mixture was adjusted to pH 7.0, filtered through 0.22 μm membranes and sterilised by ultraviolet irradiation to generate the MAP crystallisation precursor solution. A 25% ammonia solution was diluted to 5 M, sterilised by ultraviolet irradiation and stored protected from light for use as the alkalinising solution to initiate MAP crystallisation reactions.

### 2.12. In Vitro Crystal Nucleation Assay

Canine neutrophils were seeded into 24-well culture plates at a density of 5 × 10^5^ cells per well in 1 mL RPMI-1640 medium and incubated for 30 min at 37 °C in a humidified atmosphere containing 5% CO_2_. Cells were then allocated to the following treatment groups: (1) non-alkaline control; (2) non-alkaline PMA; (3) alkaline control; and (4) alkaline PMA. Neutrophils in the PMA groups were stimulated with 400 nM PMA, whereas control groups received an equal volume of RPMI-1640 medium. After 4 h of incubation to induce NET formation, 1 mL of MAP crystallisation precursor solution was added to each well.

For the alkaline groups, 50 μL of 5 M ammonia solution was added to each well to adjust the final reaction pH to >8.5. The alkaline pH (>8.5) was selected to mimic urease-mediated urinary alkalinization observed in infection-associated struvite urolithiasis. For non-alkaline groups, an equal volume of physiological saline was added. After standing for 30 min, crystal formation was examined under a light microscope (Leica, Wetzlar, Germany). Fifteen random fields per group were selected at 4× magnification for quantitative analysis of MAP crystal numbers.

### 2.13. In Vitro Crystal Aggregation Assay

Canine neutrophils were seeded into 24-well culture plates at a density of 5 × 10^5^ cells per well. After PMA and DNase I treatments for 4 h as described in Section 2.7, culture supernatants were removed.

For the struvite mineral ion system, 1 mL of MAP crystallisation precursor solution was added to each well, followed by the addition of 50 μL of 5 M ammonia solution to induce MAP crystal formation. For the calcium oxalate mineral ion system, 500 μL of 10 mM CaCl_2_ solution and 500 μL of 1 mM Na_2_C_2_O_4_ solution were added to each well to induce CaOx crystal formation.

After gentle mixing, plates were incubated in a humidified incubator for an additional 6 h. Crystal aggregation and the formation of crystal–NET complexes were subsequently examined by light microscopy (Leica, Wetzlar, Germany).

### 2.14. In Vitro Stone Growth Assay

Cryopreserved urolith specimens were ultrasonically cleaned for 40 min, sterilised by autoclaving and dried at 46 °C for at least 6 h. Each stone was weighed three times and the mean value was recorded as the initial mass (m_1_). After ultraviolet irradiation for 30 min, stones were used for subsequent experiments.

Isolated neutrophils were seeded into culture plates at a density of 5 × 10^5^ cells per well and treated as described in Section 2.7. After 30 min of incubation, one pre-treated struvite or CaOx urolith was placed into each well and incubated at 37 °C in a humidified atmosphere containing 5% CO_2_ for 6 h. Culture supernatants were then removed and replaced with the MAP or CaOx mineral ion crystallisation systems described in Section 2.11, corresponding to the urolith type, and cultures were maintained for 14 days. During the incubation period, plates were gently agitated for 1 min every 24 h to simulate urinary flow conditions.

At the end of the incubation period, uroliths were retrieved, dried at 46 °C for at least 6 h and weighed three times to obtain the mean final mass (m_2_). The percentage increase in stone mass was calculated as follows:

Stone weight gain (%) = (m_2_ − m_1_)/m_1_ × 100%.

### 2.15. Statistical Analysis

All statistical analyses were performed using SPSS 30.0 and GraphPad Prism 9.0 software. Data were assessed for normality prior to analysis. Normally distributed data are presented as mean ± standard deviation (SD) unless otherwise specified. Non-normally distributed data are expressed as median with interquartile range (IQR).

Comparisons between two groups were performed using two-tailed unpaired Student’s *t*-tests for normally distributed data and Mann–Whitney U tests for non-normally distributed data. Comparisons among multiple groups were analysed by one-way analysis of variance (ANOVA) followed by Tukey’s post hoc multiple-comparison test; when data did not meet normality assumptions, the Kruskal–Wallis test followed by Dunn’s post hoc test was applied. For datasets involving two independent variables, two-way ANOVA was performed with appropriate multiple-comparison corrections. Correlations were assessed using Pearson’s correlation analysis. A *p* value < 0.05 was considered statistically significant.

## 3. Results

### 3.1. NET-Associated Markers Are Present in Uroliths and Urine of Dogs with Urolithiasis

**Figure 1:**
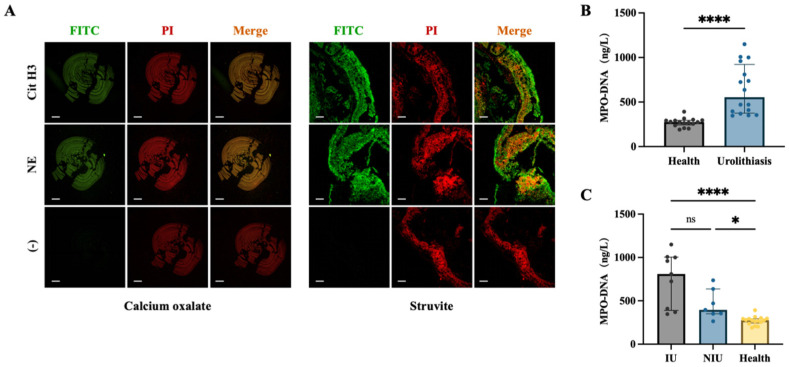
**NET-associated markers are present in uroliths and urine of dogs with urolithiasis.** (**A**) Representative immunofluorescence images showing extracellular DNA (ecDNA), citrullinated histone H3 (Cit-H3) and neutrophil elastase (NE) in canine calcium oxalate and struvite urolith sections. Scale bar = 300 μm. (**B**) Urinary concentrations of MPO–DNA complexes in healthy dogs (*n* = 16) and dogs with urolithiasis (*n* = 16). Data were analysed using the Mann–Whitney *U* test and are presented as median (IQR). **** *p* < 0.0001. (**C**) Urinary MPO–DNA concentrations in dogs with infectious urolithiasis (IU, *n* = 9), non-infectious urolithiasis (NIU, *n* = 7) and healthy dogs (*n* = 16). Data were analysed using the Kruskal–Wallis test and are presented as median (IQR). * *p* < 0.05, **** *p* < 0.0001; ns, not significant.

Immunofluorescence staining of urolith sections demonstrated the presence of NET-associated markers in both canine struvite and calcium oxalate uroliths. Pronounced co-localisation of citrullinated histone H3 (Cit-H3) with extracellular DNA (ecDNA), as well as neutrophil elastase (NE) with ecDNA, was observed within the urolith matrix (Figure 1A).

ELISA quantification of urinary MPO–DNA complexes revealed that dogs with either struvite or CaOx urolithiasis exhibited significantly higher urinary MPO–DNA levels compared with healthy control dogs (*p* < 0.0001; Figure 1B). Furthermore, when urolithiasis cases were stratified by infection status, urinary MPO–DNA concentrations were significantly elevated in dogs with non-infectious urolithiasis (*p* < 0.05) and were markedly increased in dogs with infection-associated urolithiasis (*p* < 0.0001) relative to healthy controls (Figure 1C). The clinical and laboratory characteristics of all dogs included in this study, including breed, age, sex, stone type, disease status, bacterial culture results, and urinary MPO–DNA concentrations, are summarized in Table S1.

### 3.2. Common Urinary Tract Pathogens Induce NET Formation

**Figure 2:**
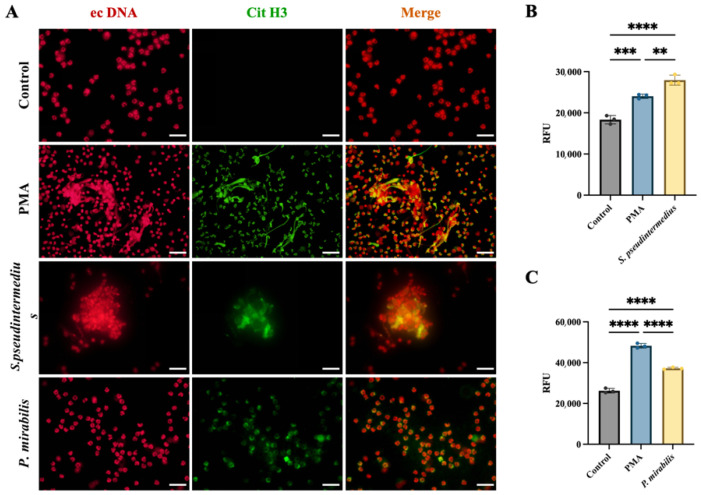
***P. mirabilis* and *S. pseudintermedius* induce NET formation in canine neutrophils.** (**A**) Representative immunofluorescence images showing ecDNA (red, propidium iodide staining) and citrullinated histone H3 (Cit-H3, green) in canine neutrophils from the control, PMA, *S. pseudintermedius* and *P. mirabilis* groups. Co-localisation of ecDNA and Cit-H3 in the merged images indicates NET formation. Scale bar = 50 μm. (**B**,**C**) Quantification of extracellular DNA release from neutrophils following stimulation with PMA or uropathogenic bacteria (*S. pseudintermedius* or *P. mirabilis*) using the Sytox Green assay. Data are presented as mean ± SD and were analysed by one-way ANOVA with multiple comparisons. *n* = 3. ** *p* < 0.01, *** *p* < 0.001, **** *p* < 0.0001.

Immunofluorescence analysis revealed weak Cit-H3 signals in untreated control neutrophils. In contrast, marked ecDNA release was observed following stimulation, with pronounced co-localisation of ecDNA and Cit-H3 forming aggregated, filamentous and punctate structures. Stimulation with *S. pseudintermedius* predominantly induced aggregated ecDNA–Cit-H3 co-localised structures, whereas stimulation with *P. mirabilis* primarily resulted in punctate ecDNA–Cit-H3 co-localised patterns (Figure 2A).

Quantitative analysis using the Sytox Green assay demonstrated that the levels of ecDNA induced by the *S. pseudintermedius* group and the *P. mirabilis* group were significantly higher than those in the control group (*p* < 0.0001) (Figure 2B,C). Furthermore, the fluorescence intensity in the *S. pseudintermedius* group was significantly higher than that in the PMA group (*p* < 0.01) (Figure 2C).

### 3.3. NETs Function as Biomineralisation Scaffolds That Drive Crystal Formation, Aggregation and Stone Growth In Vitro

**Figure 3:**
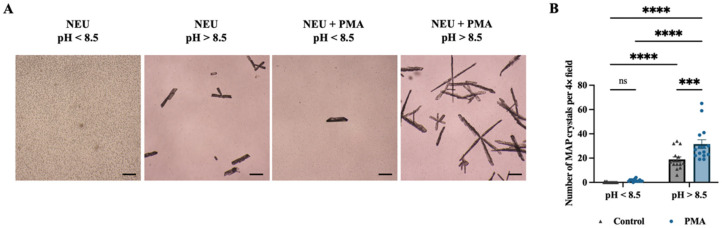
**NETs synergise with an alkaline microenvironment to promote struvite crystal formation.** (**A**) Representative bright-field micrographs (4×) showing MAP crystal formation under non-alkaline (pH < 8.5) and alkaline (pH > 8.5) conditions in control and PMA-induced NET groups. Scale bar = 200 μm. (**B**) Quantitative analysis of MAP crystal numbers per 4× field. Data were analysed by two-way ANOVA and are presented as mean ± SEM. *n* = 15. ns, not significant; *** *p* < 0.001, **** *p* < 0.0001.

Light microscopic examination revealed that the number of MAP crystals was markedly higher under alkaline conditions than under non-alkaline conditions (Figure 3A). Quantification of MAP crystals per field followed by two-way ANOVA demonstrated that both the pH of the struvite mineral ion solution and PMA-induced NET formation exerted significant main effects on MAP crystal numbers (both *p* < 0.0001), with a significant interaction between these two factors (*p* < 0.0001). Under alkaline conditions (pH > 8.5), the PMA-treated group exhibited significantly higher MAP crystal numbers per field compared with the corresponding control group (*p* < 0.001) (Figure 3B).

**Figure 4:**
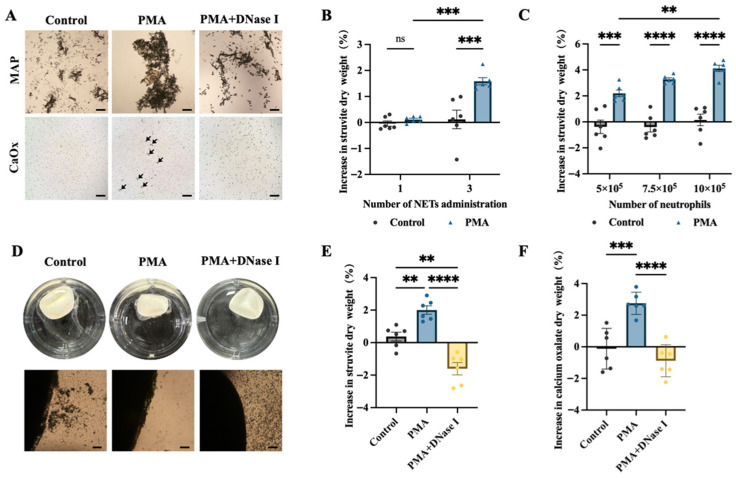
**NETs function as biomineralisation scaffolds that promote MAP and CaOx crystal aggregation and canine urolith growth.** (**A**) Representative bright-field micrographs showing aggregation of MAP and CaOx crystals. The arrows indicate CaOx crystal-NET complexes. Scale bar = 200 μm. (**B**) Percentage weight gain of struvite under different frequencies of NET exposure. Data were analysed by two-way ANOVA and are presented as mean ± SEM. *n* = 6. ns, not significant; *** *p* < 0.001. (**C**) Quantitative analysis of struvite weight gain under different neutrophil densities. Data were analysed by two-way ANOVA and are presented as mean ± SEM. *n* = 6. ** *p* < 0.01, *** *p* < 0.001, **** *p* < 0.0001. (**D**) Representative gross images of struvite after 14 days of in vitro culture and corresponding surface crystal deposition. Scale bar = 200 μm. (**E**,**F**) Percentage weight gain of struvite (**E**) and CaOx (**F**) uroliths following PMA-induced NET formation and DNase I-mediated NET degradation. Data were analysed by one-way ANOVA and are presented as mean ± SD. *n* = 6. ** *p* < 0.01, *** *p* < 0.001, **** *p* < 0.0001.

In both MAP and CaOx crystallisation systems, pronounced crystal aggregation and the formation of crystal–NET complexes were observed in the PMA group, whereas these composite structures were markedly reduced following DNase I treatment (Figure 4A).

To assess the effect of repeated NET exposure on stone growth, stone weight gain percentages after 14 days of culture were compared. No significant difference in stone weight gain was observed between the single-exposure NET group and its control (*p* > 0.05). In contrast, under conditions of repeated NET exposure (three cycles), struvite uroliths in the NET-treated group exhibited a significantly higher percentage weight gain than controls (*p* < 0.001) (Figure 4B).

To evaluate the effect of neutrophil abundance on stone growth, urolith growth assays were performed using increasing neutrophil densities (5 × 10^5^, 7.5 × 10^5^ and 10 × 10^5^ cells per well). Stone weight gain increased in a dose-dependent manner with increasing neutrophil numbers (*p* < 0.001) (Figure 4C), and stone weight gain was strongly positively correlated with neutrophil number (Pearson r = 0.997, *p* < 0.05).

In stone growth assays, neutrophils in control cultures transiently adhered to the stone surface but were poorly retained and readily detached upon agitation. In contrast, PMA-induced NET-treated cultures exhibited stable crystal and cell adhesion to the stone surface. Under long-term co-culture conditions, NETs persistently enveloped and adhered to the stone surface, and the resulting NET–crystal composite structures remained resistant to vigorous agitation. Following DNase I treatment, cellular structures were no longer detectable and crystals were predominantly dispersed (Figure 4D).

Quantitative analysis of stone weight gain demonstrated that for both struvite (Figure 4E) and CaOx (Figure 4F) uroliths, PMA-induced NET treatment resulted in significantly higher stone weight gain compared with controls, whereas DNase I treatment significantly attenuated stone growth (*p* < 0.001).

## 4. Discussion

From the perspective of innate immunity, this study demonstrates that NETs may function as endogenous biomineralization scaffolds in canine urolithiasis. NETs appear to participate in stone formation and growth under the experimental conditions examined. These findings provide new insights into the mechanisms underlying urinary stone development and their high recurrence rate in dogs.

Multiple factors can trigger NET formation in canine urolithiasis. In healthy individuals, NETosing neutrophils and low levels of NETs can already be detected in urine, indicating that the urinary tract possesses an innate immune defense mechanism that can be rapidly activated [36]. Consistent with this concept, we found that urinary MPO–DNA levels were significantly higher in dogs with urolithiasis than in healthy controls, indicating sustained NET activation in the urolithiasis microenvironment. Previous studies have shown that various endogenous stimuli can induce NETosis, including immune complexes [37], activated platelets [38], and inflammatory cytokines [39,40]. Urinary stones persist in the urinary tract for prolonged periods and can continuously provoke local chronic inflammation [41]. This leads to the accumulation of endogenous stimulatory factors within the local microenvironment. Therefore, stone-associated chronic inflammation can promote persistent NETosis through multiple endogenous activation pathways [30,42].

Pathogenic microorganisms may represent key triggers of NETosis. In this study, extremely high levels of urinary MPO–DNA complexes were detected in one dog with struvite urolithiasis complicated by *S. pseudintermedius* infection and in one dog with calcium oxalate urolithiasis complicated by *P. mirabilis* infection. These findings indicate that NETs are excessively activated under infectious conditions. Previous studies have also reported upregulation of NET-associated proteins in infection-related stones, supporting the sustained presence of NETs within the infectious stone microenvironment [22]. Together, these observations suggest that urinary tract pathogens may serve as important drivers of excessive local NET release.

*S. pseudintermedius* and *P. mirabilis* are common uropathogens in dogs [7,12,43,44]. Our in vitro experiments further demonstrated that both bacteria can directly induce NETosis in canine peripheral blood neutrophils, establishing a “pathogen–neutrophil–NETs” immune axis. This finding provides a plausible biological explanation for a clinically important observation: urolithiasis patients with concurrent urinary tract infection exhibit significantly higher recurrence rates [45,46]. Notably, this phenomenon is not restricted to classical infection-associated struvite stones but is also observed in non-infectious stones [46]. Therefore, urinary tract infection promotes stone formation and progression not only through urease-mediated urine alkalinization.

NETs are critical contributors to pathological mineralization. NETs are fibrous, web-like extracellular structures composed of a DNA backbone decorated with histones and granular proteins [28]. The DNA scaffold is negatively charged and facilitates the attraction and stabilization of cations, whereas many NET-associated proteins carry positive charges and can bind anions [47,48]. When local ionic concentrations exceed saturation thresholds, NETs provide favorable nucleation sites that promote crystal precipitation [48]. Our results further support the concept that NETs function as structural scaffolds for ectopic mineralization. NETs promoted MAP crystal formation, with a more pronounced effect under alkaline conditions. In addition, NETs possess a porous reticular architecture with a mean pore area of 0.03 ± 0.04 μm^2^ [49]. While this structure physiologically serves to trap microbes and inflammatory mediators to limit their dissemination [50], our findings suggest that the same NET framework may also capture MAP and CaOx crystals. In this context, NETs may act as a “biological glue” that continuously recruits additional crystals and ultimately forms stable NET–crystal aggregates.

Stone formation is a prolonged and cumulative process. In this study, a significant increase in stone weight was observed only after repeated exposure to NETs, indicating that canine urolith growth depends on sustained rather than single NETs stimulation. This finding conceptually aligns with the chronic and recurrent inflammatory conditions frequently observed in clinical cases of urolithiasis, highlighting the potential role of persistent NET-associated inflammation in promoting progressive stone enlargement. Consistent with this, decalcified stone sections displayed an “onion-like” layered distribution of ecDNA and Cit-H3, showing periodic stacking within the stone matrix. These features suggest that NETs participate in early nucleation and continuously mediate subsequent mineralization and stone growth. Moreover, stronger Cit-H3 and NE fluorescence signals were detected in the outermost layers of struvite stones, and NETs were observed to adhere to crystal deposits on stone surfaces. Together, these findings indicate that newly formed stone layers are primarily driven by persistent NETs in the local microenvironment. This pattern is consistent with observations in other pathological calcification disorders and further supports a structural role for NETs in sustained stone growth [35,40].

Urine is a metabolically generated biological fluid composed primarily of water, inorganic ions, and diverse organic components. In canine urolithiasis, crystal formation is driven not only by ionic supersaturation but also by inflammatory processes within the urinary tract. To dissect these interrelated factors, we established two complementary in vitro systems: a supersaturated mineral ion system to represent the physicochemical contribution of inorganic components, and a NET-based cellular model to represent inflammation-associated organic scaffolding within the urinary microenvironment. In vivo, urinary ionic concentrations and pH values fluctuate dynamically under the influence of hydration status, infection, metabolic activity, and urinary flow. Building upon previously reported mineral ion systems, we optimized and established a modified ionic supersaturation range and pathologically relevant pH conditions that permitted crystal formation while preserving neutrophil viability. This allowed objective evaluation of nucleation and aggregation processes under controlled experimental conditions. Regarding NET induction, NETosis in vivo is triggered by multifactorial inflammatory signals, including bacterial products, chemokines, and immune cell interactions. In this study, PMA was employed as a standardized and reproducible stimulus, and stimulation conditions were optimized in preliminary experiments to ensure stable NET formation. This strategy enabled focused investigation of the structural contribution of NET-derived extracellular chromatin to crystal growth.

This study has several limitations. First, the sample size of certain urinary subgroups was relatively limited, which may constrain the generalizability of subgroup-specific findings. Larger-scale and multicenter investigations will be necessary to further validate these associations. Second, the inherent structural heterogeneity of uroliths presents methodological challenges for standardized quantitative comparison of fluorescence intensity across samples. Future layer-specific analyses may provide more precise insights into the spatial distribution of NETs within distinct stone regions. In addition, although the in vitro mineral ion system and standardized PMA-induced NET model captured essential mechanistic aspects of crystal–NET interactions, they do not fully reproduce the dynamic urinary microenvironment in vivo. These systems should therefore be interpreted as controlled mechanistic platforms rather than comprehensive physiological simulations. Finally, while DNase I attenuated NET-associated crystal growth under in vitro conditions, its therapeutic applicability remains speculative and warrants further validation in animal models before clinical translation.

## 5. Conclusions

Traditional theories attribute canine urolith formation mainly to urinary supersaturation, pH alteration, and infection. Our findings identify a previously unrecognized mechanism in which NETs act as DNA-based biomineralization scaffolds that promote crystal nucleation, aggregation, and stone growth. The promotive effect of NETs on stone formation observed under in vitro conditions may partly explain the high recurrence rate and persistent progression of urinary stones. Therefore, targeting NET formation may represent a potential adjunct avenue for future investigation.

## Data Availability

The original contributions presented in this study are included in the article. Further inquiries can be directed to the corresponding authors.

## Supplementary Materials

The following supporting information can be downloaded at: https://www.mdpi.com/article/10.3390/ani16060942/s1, Table S1: Breed, age, sex, stone type, condition of disease, bacterial culture result, and urinary MPO-DNA concentration of all dogs were included in this study.
